# Association between ambient air pollution and cause-specific mortality in Cape Town, Durban, and Johannesburg, South Africa: any susceptible groups?

**DOI:** 10.1007/s11356-021-13778-w

**Published:** 2021-04-07

**Authors:** Nomsa Duduzile Lina Thabethe, Kuku Voyi, Janine Wichmann

**Affiliations:** 1 Department of Environmental Sciences, University of South Africa, 28 Pioneer Street, Florida, 1709 South Africa; 2 School of Health Systems and Public Health, Health Sciences Faculty, University of Pretoria, P.O. Box 667, Pretoria, 0001 South Africa

**Keywords:** Ambient air pollution, Susceptibility, Cause-specific mortality, Respiratory diseases, Cardiovascular diseases

## Abstract

Studies have confirmed that adverse human health effects that are associated with exposure to air pollution may differ depending on other factors such as age, gender, environmental conditions, and socio-economic factors. This study was conducted to assess the association between ambient air pollution and cause-specific mortality in the three big cities in South Africa and to determine the susceptible groups thereof. Cause-specific mortality data for all ages and PM_10_, NO_2_, and SO_2_ in Cape Town, Durban, and Johannesburg for the period from 1 January 2006 to 31 December 2010 were obtained. Statistical analyses were done to estimate the associations between air pollutants and cause-specific mortality. Susceptibility was therefore investigated in stratified analyses by sex and age (≥60 years) and environmental conditions (heat and cold) followed by models with interaction terms. Our estimates showed independent associations between these air pollutants, environmental conditions, and susceptible groups.

## Introduction

Air pollution has detrimental impacts on human health (Liu et al., 2019, b; Khaniabadi et al. 2019; Gu et al. 2019) with health effects ranging from cardiovascular diseases (CVD) (Curto et al. 2019), respiratory diseases (RD) (Croft et al. 2019), and mortality (Chen et al. 2019). The increase in the levels of ambient air pollution is associated with increased adverse health effects (Trinh et al. 2019; Chen et al. 2019); and there is documented evidence that decreased exposure to air pollution decreases population mortality and morbidity and it increases life expectancy (Brønnum-Hansen, et al. 2018; Hoffmann 2019). Increases in air pollution levels are associated with increases in health effects of susceptible groups such as children, the elderly, pregnant women, and people with chronic diseases (Stieb et al. 2019; Koman et al. 2018; Hooper et al. 2018).

Children are susceptible to negative health impacts caused by air pollution due to their underdeveloped pulmonary and immune systems and smaller airways that become more obstructed when inflamed (Salvi 2007). In addition to this, children have higher levels of outdoor physical activity as compared to adults (Alhanti et al. 2016). On the other hand, the elderly is also susceptible to the effects of air pollution because of their reduced lung function that occurs as a natural part of aging (Viegi et al. 2009). Pre-existing diseases in the elderly and other factors also make them more susceptible to the health effects of air pollution (Karimi and Samadi 2019).

Epidemiological studies have shown that CVD (Ho et al. 2020; Khaniabadi et al. 2019; Dastoorpoor et al. 2019), RD (Ho et al. 2020; Karimi and Samadi 2019), and climatic conditions (Lou et al. 2019; Scortichini et al. 2018) are associated with the cause of morbidity and mortality, more especially in susceptible groups. As much as there is evidence of association between these confounders and air pollution mortality, these studies did not investigate how much of an impact does these confounders have on the susceptible groups living in South Africa.

This study addresses these limitations by estimating associations between air pollution levels and cause specific mortality in three large cities in South Africa taking into consideration the different ages of the population and the climatic conditions.

## Methods

The association between 24-h average outdoor air pollution levels (PM_10_, NO_2_, SO_2_) and CVD and RD deaths were investigated with the time-stratified case-crossover epidemiological study design.

The cause-specific mortality data (all ages) for Cape Town, Durban, and Johannesburg for the study period 1 January 2006–31 December 2010 were obtained from the Health and Vital Statistics Division, Statistics South Africa. RD deaths were those with 10th Version of the International Classification of Diseases (ICD10) codes J00-J999 and CVD deaths those with codes I00-I52.

The air pollution data for Cape Town, Durban, and Johannesburg for the period 1 January 2006–31 December 2010 were obtained from the South African Weather Services (SAWS) as 1-h averages. SAWS manages the South African Air Quality Information System (SAAQIS). Daily 24-h averages (midnight-to-midnight) of a pollutant measured at the selected monitoring sites were calculated from the hourly data and were based on at least 18 1-h values in accordance with the ISO 17025 guidelines. Then an aggregated 24-h average for each of the selected pollutants was calculated across the entire city. At the time of the study, PM_2.5_ was not monitored in the three cities. The daily and yearly PM_2.5_ South African air quality standard only came into effect on 29 June 2012 (South Africa 2009).

The temperature (°C) and relative humidity (%) data for Cape Town, Durban, and Johannesburg for the period 1 January 2006–31 December 2010 were obtained from SAWS as 1-h averages. Daily 24-h averages (midnight-to-midnight) were calculated from the hourly data and were based on at least 18 1-h values.

According to Barnett et al. (2010), there is no single temperature measure that is superior to others. In this study, temperature and relative humidity were adjusted for as apparent temperature (Tapp), which is a construct intended to reflect the physiological experience of combined exposure to humidity and temperature and thereby better capture the response on health than temperature alone (Steadman 1984; Wichmann and Voyi 2012).

A time-stratified approach was applied to select the control days, defining the day of death as the case day and the same day of the other weeks in the same month and year as control days. With this approach, even very strong confounding of exposure by seasonal patterns is controlled by design (Carracedo-Martíne et al. 2010; Wichmann and Voyi 2012).

Influenza data were not available on city level. We used the method from a large European study to control for influenza by including a binary variable taking the value of one when the 7-day moving average of the RD mortality was greater than the 90^th^ percentile of its city-specific distribution. Because the proxy variable for influenza was based on the distribution of RD mortality, it was not included in the RD mortality models, only in the CVD or CBD mortality models.

The associations between the 2-day cumulative average of lag0 and lag1 of the air pollutants and mortality were investigated; as done in previous studies (Shah et al. 2015). Lag0 refers to the air pollution concentration on the day of death and lag1 to the concentration the day before death.

Previous studies reported a linear relationship between PM_10_, NO_2_, and SO_2_ and the cause-specific deaths (Sacks et al. 2012). These pollutants were therefore included as linear terms in the models, one pollutant at a time (i.e., single-pollutant models). In order to disentangle the health outcomes (e.g., mortality) attributed to individual pollutants, previous researchers have used co-pollutant or multi-pollutant models. However, the interpretation of results from these models is complicated because regression models become highly unstable when incorporating pollutants that are highly correlated (Dominici et al. 2010; Sacks et al. 2012). The associations were investigated using conditional logistic regression models (PROC PHREG in SAS 9.2, SAS Institute, Cary, NC). Models were adjusted for public holidays (binary variable) and the 2-day cumulative average of Tapp.

Odds ratios (OR) and the 95% confidence intervals (CI) were calculated per inter-quartile range (IQR) increase in the pollutant levels, which provided magnitude-of-risk estimates that were comparable across the exposure variables. The results were presented as the percent excess risk in cause-specific deaths per IQR increase in a pollutant using the following calculation: (exp^(βxIQR)^ – 1) × 100%, where *β* is the model estimate (Wichmann and Voyi 2012).

Susceptibility was investigated in stratified analyses by sex and age (all ages and ≥60 years), followed by models with interaction terms. Models were run separately for each city and each cause-specific mortality. The number of RD and CVD deaths was small in the 0–4-year group, so models were not run for this age group.

In the meta-analysis, the heterogeneity of the air pollutant associations with RD and CVD mortality in the three cities was assessed for all ages combined. Meta-analyses were not conducted on the associations observed for the ≥60 year groups as the associations for these age groups did not differ significantly from all ages combined. A fixed-effect model was applied to summarize the pooled estimates as the *Q* and *I*^2^ test statistics for heterogeneity were non-significant. The meta package of the R statistical software was applied (R Development Core Team, 2016).

## Results

### Descriptive statistics

Table 1 displays the descriptive statistics of the PM_10_, NO_2_, and SO_2_ levels and the meteorological conditions in the three cities. On average the daily mean concentrations of PM_10_, NO_2_, and SO_2_ in Cape Town were 32.7, 17.5, and 10.4 μg/m^3^, respectively, during the 5-year study period. The yearly mean PM_10_ level in Cape Town exceeded the more protective yearly WHO guideline (20 μg/m^3^) during all 5 years, but never exceeded the more lenient yearly South African standard (75 μg/m^3^). The yearly mean NO_2_ level in Cape Town never exceeded the yearly WHO guideline (40 μg/m^3^) during all 5 years. The yearly NO_2_ South African standard is also 40 μg/m^3^. The yearly mean SO_2_ level in Cape Town never exceeded the yearly South African standard (50 μg/m^3^). There is no yearly SO_2_ WHO guideline.

**Table 1:** Descriptive statistics for daily NO_2_, SO_2_, and PM_10_ levels and meteorological conditions in Cape Town, Durban, and Johannesburg, South Africa, during 1 January 2006 to 31 December 2006

	Mean	Std dev.	Min	Max	Percentiles		
					25th	50th	75th
Cape Town							
PM_10_ (μg/m^3^)	32.8	14.6	7.9	121.5	22.2	29.8	41.3
NO_2_ (μg/m^3^)	17.5	8.7	3.4	59.8	11.2	15.6	22.2
SO_2_ (μg/m^3^)	10.4	6.4	0.8	53.5	6.0	8.8	13.2
Tapp (°C)	15.6	4.4	5.4	29.3	12.1	15.5	19.1
Temperature (°C)	17.0	3.7	8.0	29.0	14.0	17.0	20.0
Relative humidity (%)	74.1	10.2	37.0	98.0	67.0	75.0	81.0
Wind speed (m/s)	5.1	2.2	1.0	13.0	4.0	5.0	7.0
Rain (mm)	1.4	4.2	0.0	49.0	0.0	0.0	0.0
Durban							
PM_10_ (μg/m^3^)	32.2	19.2	5.8	146.4	19.5	27.1	38.4
NO_2_ (μg/m^3^)	33.2	14.8	9.9	131.1	23.1	30.1	39.6
SO_2_ (μg/m^3^)	20.3	10.3	3.1	76.9	12.8	18.1	25.7
Tapp (°C)	20.8	4.3	9.9	30.8	17.5	20.8	24.1
Temperature (°C)	21.0	3.1	12.0	28.0	19.0	21.0	23.0
Relative humidity (%)	75.6	8.8	36.0	97.0	71.0	77.0	81.0
Wind speed (m/s)	3.9	1.5	1.0	11.0	3.0	4.0	5.0
Rain (mm)	2.7	8.4	0.0	144.0	0.0	0.0	1.0
Johannesburg							
PM_10_ (μg/m^3^)	57.3	27.5	7.7	273.3	37.4	51.0	72.5
NO_2_ (μg/m^3^)	51.9	20.9	0.9	123.1	37.3	50.8	64.4
SO_2_ (μg/m^3^)	16.9	13.5	1.2	90.7	6.9	13.0	23.3
Tapp (°C)	14.5	4.7	0.0	24.0	10.9	15.3	18.2
Temperature (°C)	16.4	4.3	2.0	26.0	13.0	17.0	20.0
Relative humidity (%)	56.1	19.2	10.0	98.0	41.0	57.0	71.0
Wind speed (m/s)	4.1	1.4	0.0	9.0	3.0	4.0	5.0
Rain (mm)	1.9	5.7	0.0	65.0	0.0	0.0	0.0

The daily PM_10_ levels in Cape Town exceeded the more protective daily WHO guideline (50 μg/m^3^) on 200 days during the study period, compared to only 20 days when compared to the more lenient daily South African standard (75 μg/m^3^). The daily SO_2_ levels in Cape Town exceeded the more protective daily WHO guideline (20 μg/m^3^) on 114 days during the study period and never exceeded the more lenient daily South African standard (125 μg/m^3^).

On average the daily mean concentrations of PM_10_, NO_2_, and SO_2_ in Durban were 32.2, 33.2, and 20.3 μg/m^3^, respectively, during the 5-year study period. The yearly mean PM_10_ level in Durban exceeded the more protective yearly WHO guideline during all 5 years, but never exceeded the more lenient yearly South African standard. The yearly mean NO_2_ level in Durban never exceeded the yearly WHO guideline or the yearly South African standard. The yearly mean SO_2_ level in Durban never exceeded the yearly South African standard.

The daily PM_10_ levels in Durban exceeded the more protective daily WHO guideline on 207 days during the study period, compared to only 57 days when compared to the more lenient daily South African standard. The daily SO_2_ levels in Durban exceeded the more protective daily WHO guideline on 725 days during the study period and never exceeded the more lenient daily South African standard.

On average the daily mean concentrations of PM_10_, NO_2_, and SO_2_ in Johannesburg were 57.3, 51.9, and 16.9 μg/m^3^, respectively, during the 5-year study period. The yearly mean PM_10_ level in Johannesburg exceeded the more protective yearly WHO guideline during all 5 years, but never exceeded the more lenient yearly South African standard. The yearly mean of SO_2_ level in Johannesburg never exceeded the yearly South African standard. The yearly mean SO_2_ level in Johannesburg never exceeded the yearly South African standard.

The daily PM_10_ levels in Johannesburg exceeded the more protective daily WHO guideline on 807 days during the study period, compared to 359 days when compared to the more lenient daily South African standard. The daily SO_2_ levels in Johannesburg exceeded the more protective daily WHO guideline on 270 days during the study period and never exceeded the more lenient daily South African standard.

Of the three cities, the Durban had the highest mean Tapp during the study period (20.8 °C), followed by Cape Town (15.6 °C) and Johannesburg (14.5 °C). Cape Town was the windiest of the three cities. Durban received more rain than Johannesburg or Durban.

**Fig. 1:**
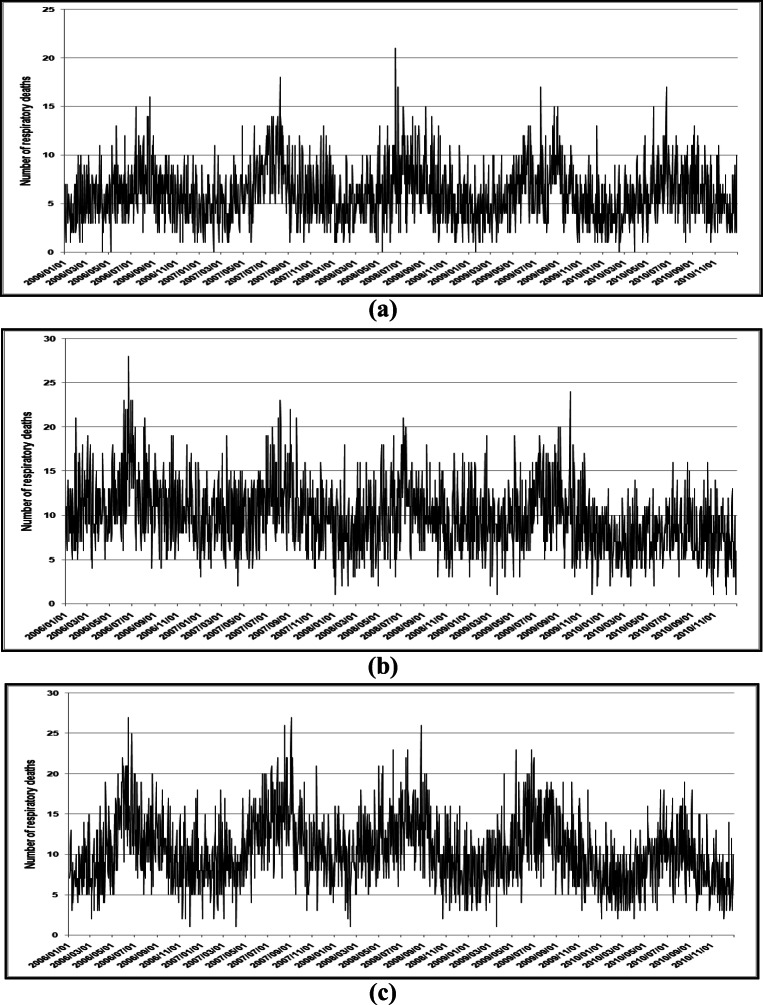
Time series of respiratory disease mortality in **a** Cape Town, **b** Durban, and **c** Johannesburg during January 2006–December 2010

**Fig. 2:**
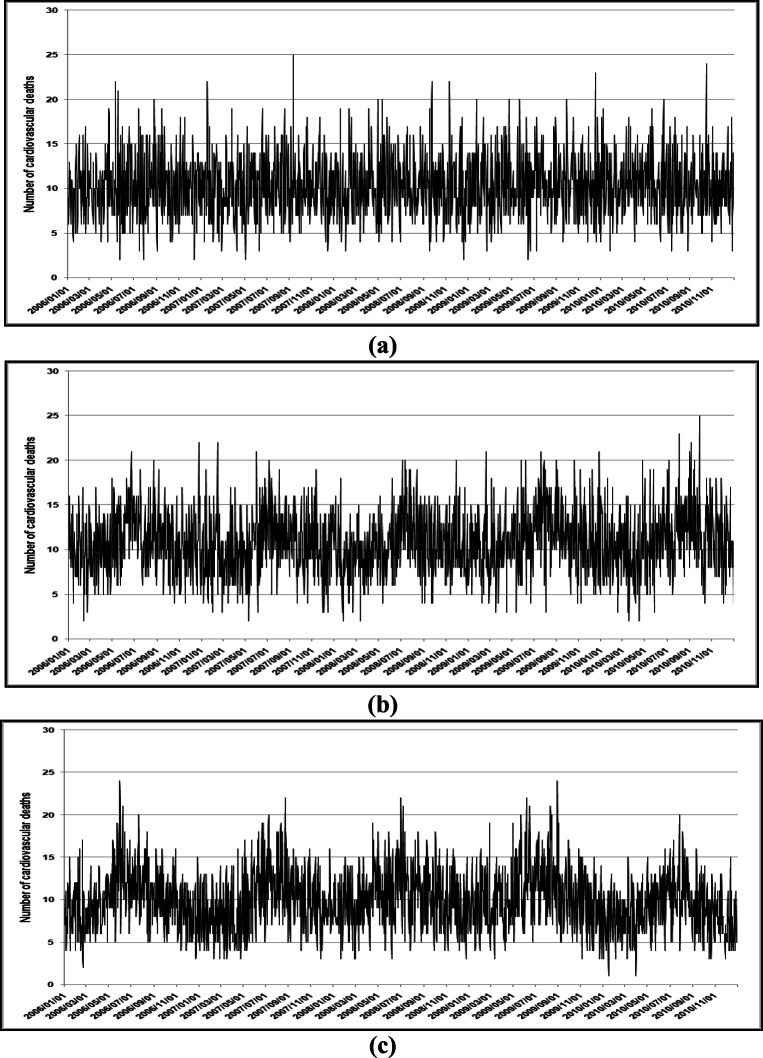
Time series of cardiovascular disease mortality in **a** Cape Town, **b** Durban, and **c** Johannesburg during January 2006–December 2010

Figure 1 illustrates the time-series of RD mortality in Cape Town, Durban, and Johannesburg during January 2006 to December 2010. Typical seasonal trends are observed with more RD deaths during the colder than warmer months. Johannesburg had the highest number of RD deaths, followed by Durban and Cape Town (Table 2). In all three cities, more RD deaths occurred among the elderly (≥60 years). Cape Town had the highest number of RD deaths in the ≥60 year age group (Fig. 2). Durban had the highest number of CVD deaths, followed by Cape Town and Durban (Table 2). As with RD deaths, in all three cities, more CVD deaths occurred among the elderly than children. Cape Town had the highest number of RD deaths in the ≥60 year age group.

**Table 2:** Number of respiratory and cardiovascular disease deaths in Cape Town, Durban, and Johannesburg during 1 January 2006 to 31 December 2010

City	Age groups	Respiratory diseases	Cardiovascular diseases
Cape Town	All ages	10,936	18,822
	0–4 years	871	132
	≥60 years	6000	13,485
	Missing	9	6
Durban	All ages	17,893	19,810
	0–4 years	2028	179
	≥60 years	4916	12,218
	Missing	51	13
Johannesburg	All ages	18,847	17,984
	0–4 years	2069	275
	≥60 years	5962	10,659
	Missing	106	34

Population during Census 2011: Cape Town, 3,740,026; Durban, 3,442,361; Johannesburg, 4,434,827 (Statistics South Africa 2011). Population size by age groups for each city is not available from the Census 2011. Hence, rates by age groups cannot be calculated.

### Associations between air pollutants and respiratory diseases mortality

In Cape Town (Table 3), an inter-quartile range (IQR) of 17 μg/m^3^ increase in the CA2 (2-day cumulative average) of PM_10_ increased RD mortality for all ages by 0.8% (95% CI: −2.5 to 4.1%). The association between PM_10_ and RD mortality was stronger for women than men.

**Table 3:** Percentage change (95% CI) in respiratory and cardiovascular disease mortality per inter-quartile increase in the 2-day cumulative average of PM_10_, NO_2_, and SO_2_ (in μg/m^3^) from 1 January 2006 to 31 December 2010 in Cape Town

Pollutant	Ages and sex	Respiratory disease*				Cardiovascular disease**			
		IQR	%	95% CI		IQR	%	95% CI	
PM_10_	All ages	17	0.8	−2.5	4.1	17	3.5	0.9	6.1
	≥60 years	18	3.8	−0.8	8.7	17	3.8	0.7	6.9•
	Male	17	−1.9	−6.2	2.6	18	2.6	−1.2	6.6
	Female	17	3.6	−1.3	8.7	17	4.5	0.8	8.2•
NO_2_	All ages	10	1.4	−3.0	6.0	11	2.1	−1.8	6.1
	≥60 years	11	7.8	0.9	15.1#	11	1.9	−2.6	6.7
	Male	10	0.3	−5.5	6.5	11	0.4	−5.0	6.0
	Female	10	2.4	−4.2	9.5	11	0.4	−5.0	6.0
SO_2_	All ages	7	−0.2	−4.2	4.1	7	0.9	−2.2	4.1
	≥60 years	7	2.2	−3.3	8.0	7	2.4	−1.3	6.3
	Male	7	0.1	−5.3	5.8	7	−0.2	−4.7	4.4
	Female	7	−1.1	−7.1	5.3	7	2.0	−2.4	6.6

*Adjusted for apparent temperature (Tappca2) and public holidays

**Adjusted for apparent temperature (Tappca2), public holidays, and influenza

•Insignificantly different from unstratified (all ages) analysis

#Non-significant different from unstratified (all ages) analysis

In Durban (Table 4), an IQR of 19 μg/m^3^ increase in the CA2 of PM_10_ increased RD mortality by 4.4% (95% CI: −13.0 to 25.2%) in the ≥60 year group, respectively. An IQR of 17 μg/m^3^ increase in the CA2 of NO_2_ increased RD mortality by 10% (95% CI: −2.1 to 4.1%) for all ages; by 0.1% (95% CI: −4.0 to 4.5%) for males and by 1.6% (95% CI: −2.8 to 6.2%) for females.

**Table 4:** Percentage change (95% CI) in respiratory and cardiovascular disease mortality per inter-quartile increase in the 2-day cumulative average of PM_10_, NO_2_, and SO_2_ (in μg/m^3^) from 1 January 2006 to 31 December 2010 in Durban

Pollutant	Ages and sex	Respiratory disease*				Cardiovascular disease**			
		IQR	%	95% CI		IQR	%	95% CI	
PM_10_	All ages	19	0.5	−2.0	3.1	20	0.7	−1.9	3.4
	≥60 years	19	1.5	−3.2	6.5	20	−1.0	−4.2	2.3
	Male	19	0.0	−3.5	3.6	20	−1.7	−5.3	1.9
	Female	19	1.0	−2.6	4.8	20	3.3	−0.4	7.1
NO_2_	All ages	17	1.0	−2.1	4.1	17	1.7	−1.1	4.7
	≥60 years	17	2.8	−2.9	8.7	17	0.3	−3.3	4.0
	Male	17	0.1	−4.0	4.5	17	0.2	−3.8	4.3
	Female	17	1.6	−2.8	6.2	17	3.4	−0.7	7.6
SO_2_	All ages	11	−2.2	−4.8	0.4	11	1.1	−1.4	3.7
	≥60 years	11	−3.0	−7.8	1.9	11	−0.3	−3.5	2.9
	Male	11	−2.6	−6.2	1.0	11	0.0	−3.5	3.6
	Female	11	−1.8	−5.5	2.1	11	2.3	−1.2	6.1

*Adjusted for apparent temperature (Tappca2) and public holidays

**Adjusted for apparent temperature (Tappca2), public holidays, and influenza

In Johannesburg (Table 5), an IQR of 17 μg/m^3^ increase in the CA2 of PM_10_ significantly increased RD mortality by 6.7% (95% CI: 1.4 to 12.4%) for males.

**Table 5:** Percentage change (95% CI) in respiratory and cardiovascular disease mortality per inter-quartile increase in the 2-day cumulative average of PM_10_, NO_2_, and SO_2_ (in μg/m^3^) from 1 January 2006 to 31 December 2010 in Johannesburg

Pollutant	Ages and sex	Respiratory disease*				Cardiovascular disease**			
		IQR	%	95% CI		IQR	%	95% CI	
PM_10_	All ages	35	1.4	−2.2	5.1	35	3.3	−0.5	7.3
	≥60 years	37	−1.4	−7.8	5.3	36	6.6	1.4	12.1
	Male	17	6.7	1.4	12.4#	34	3.8	−1.5	9.4
	Female	17	−4.2	−9.0	0.9	36	2.8	−2.6	8.5
NO_2_	All ages	26	6.4	−0.3	13.6#	26	1.1	−5.5	8.2
	≥60 years	25	0.96	−9.5	12.7	26	5.3	−3.5	14.8
	Male	36	13.0	3.3	23.7#	27	3.8	−10.4	9.3
	Female	34	−0.5	−9.6	9.4	26	2.8	−6.4	13.2
SO_2_	All ages	17	−3.7	−9.3	2.3	16	0.6	−5.1	6.6
	≥60 years	17	−6.9	−16	3.2	16	−2.3	−9.2	5.3
	Male	17	-1.3	-9.0	7.2	16	0.7	-7.3	9.5
	Female	17	-6.5	-14.4	2.1	16	0.0	-7.9	8.6

*Adjusted for apparent temperature (Tappca2), day of the week and public holidays.

**Adjusted for apparent temperature (Tappca2), day of the week, public holidays, and influenza

#Significantly different from unstratified (all ages) analysis

### Association between air pollutants and cardiovascular disease mortality

In Cape Town (Table 3), an IQR of 17 μg/m^3^ increase in the CA2 of PM_10_ increased CVD mortality for all ages by 3.5% (95% CI: 0.9 to 6.1%). In Durban (Table 4), an IQR of 17 μg/m^3^ increase in the CA2 of NO_2_ increased CVD by 1.7% (95% CI: −1.1 to 4.7%) for all ages and by 0.3% (95% CI: −3.3 to 4.0%) for the elderly. In Johannesburg (Table 5), an IQR of 16 μg/m^3^ increase in the CA2 of SO_2_ increased CVD mortality by 0.6% (95% CI: −5.1 to 6.6%) for all ages and by 0.7% (95%CI: −7.3 to 9.5%) for males.

## Discussion

RD became one of the leading causes of deaths in the USA (Moy et al. 2017). In the three cities, Johannesburg had the highest number of RD deaths, followed by Durban and Cape Town. This is expected because Johannesburg has the biggest population compared to Cape Town and Durban. According to Statistics South Africa, Johannesburg has a population of about 4,434,827 inhabitants, Cape Town (3,740,026), and Durban (3,442,361). Johannesburg also had much higher levels of PM_10_ and NO_2_ (but not SO_2_) than Durban and Cape Town in 2006–2010. The high levels of PM_10_ could be associated with domestic fuel burning, traffic volumes, and other local sources (Czernecki et al. 2017). According to Xiao et al. (2018), the concentrations of pollutants in the atmosphere are influenced by the local sources, the temporal and spatial characteristics of the concentrations of air pollutants, and the relationship between the air pollutants and the meteorological factors. This explains the reason why there were high PM_10_ and NO_2_ concentrations and low SO_2_ levels in Johannesburg.

The highest number of RD and CVD deaths in the three cities occurred during the cold periods and when the PM_10_, NO_2_, and SO_2_ levels were high. In most cases, during warm months, when the levels of PM_10_, NO_2_, and SO_2_ decreased, the number of RD and CVD deaths also decreased. Mortality and PM_10_ levels are known to vary considerably across seasons (Li 2018). In addition, as much as cold temperatures show greater effects than hot temperatures do, factors such as respiratory epidemics make the role of temperature on increased morbidity and mortality to be unclear (Braga et al. 2002). Kim et al. (2017) found that the seasonal mortality effect of PM_10_ varied considerably by cause of death and location.

Although this study only assessed the association between RD and CVD mortality and PM_10_, NO_2_, and SO_2_ among adults who were 60 years and older, much was not done on the influence of these pollutants on gender. However, epidemiological studies suggest stronger effects of air pollution among women (Hooper et al. 2018). The explanations for this are very broad and range between biological factors related to lung volume, deposition, and reactivity and hormonal influences on chemical transport (Collins et al. 2017, Chen et al. 2017; Liu et al. 2019). Furthermore, gender explanations include confounding, smoking, alcohol abuse, exposure to chemical, and response to psychosocial stressors (Li et al. 2018). Children and the elderly are also susceptible to effects of air pollution (Kurt et al. 2016; Vrijheid et al. 2016). Children are recognized as a high-risk group, but their susceptibility may differ by childhood stages (Giorgini et al. 2016; Chuwah et al. 2017).

Our estimates showed independent associations between PM_10_, NO_2_, and SO_2_ and RD and CVD mortality (Liu et al., 2019, b). The strongest associations were seen on the day of exposure, with more constant effects for PM_10_. This could be because exposure to PM_10_ causes more harm to human health than NO_2_ and SO_2_, and for this reason, PM10 air pollution is a uniquely important public health issue among the list of novel risk factors (Johannson et al. 2015).

A major strength of this study is the high-quality of mortality and air pollution data obtained from Statistics South Africa and the South African Weather Services. Different models were used in SAS to analyze the data in order to get a wide perspective of the association between PM_10_, NO_2_, and SO_2_ and the RD and CVD.

Like all other case-crossover and time-series epidemiological studies, the limitation in this study was the assumption that the ambient air pollution and meteorological variables measured at a few sites are the same across the entire city. Another limitation is that only mortality data was used; data on effect modifiers (e.g., tobacco smoking or environmental tobacco smoke) was not available and not investigated.

## Conclusions

Our estimates of association for all age groups combined between PM_10_, NO_2_, and SO_2_; and RD and CVD mortality corresponds with other studies conducted worldwide. There was a lower number of CVD in a city that has more population and high levels of air pollution as compared to the other two cities.

## Data Availability

Not applicable.

## Declarations

This study was purely registry based, as no human participants were recruited or included in experiments. Approval from the University of Pretoria Ethics Committee to conduct a research project with the 2004 to 2008 Johannesburg mortality data was obtained (Ethics reference number 101/2013). Statistics South Africa supplied anonymous cause-specific mortality data of Cape Town, Durban, and Johannesburg for the period 1 January 2006 to 31 December 2010.
